# Uterine Rupture with Evisceration of Intestines through the Vagina during Labour

**DOI:** 10.1155/2019/5234641

**Published:** 2019-12-11

**Authors:** Ubong Akpan, Chinyere Akpanika, Victor Nwagbara, Udeme Asibong, Saturday Etuk

**Affiliations:** ^1^Department of Obstetrics and Gynaecology, University of Calabar, Calabar, Nigeria; ^2^Department of General Surgery, University of Calabar, Calabar, Nigeria; ^3^Department of Family Medicine, University of Calabar, Calabar, Nigeria

## Abstract

Uterine rupture is a life threatening obstetric emergency and is associated with high maternal and perinatal mortality. There are some risk factors associated with uterine rupture which may include: prolonged obstructed labour, previous scarred uterus, grand-multiparity, macrosomic baby, abnormal lie, instrumental delivery, induction of labour, oxytocin stimulation and excessive uterine manipulation. Its modes of presentation have been widely reported. Here, we present a case with an unusual mode of presentation where about two-third of the small intestines protruded through the vagina following some manipulations by an unskilled birth attendant. This highlights the fact that when uterine rupture is suspected, the cord-like structure protruding per vaginum may not always be umbilical cord.

## 1. Background

Transvaginal bowel evisceration is rare. To date less than 100 cases have been reported in the literature. Most of the cases reported followed ruptured vaginal vault in post hysterectomized menopausal women or sexual assault [1, 2]. It may also be caused by penetrating injury involving the posterior vaginal fornix or the Pouch of Douglass or uterine perforation following unsafe abortion [3, 4].

Intestinal prolapse through the vagina has also been reported in ulcerating gynaecological malignancies [5, 6]. Cases of intra-partum visceral prolapse per vaginum, to the best of our knowledge, have not been reported in the literature. We hereby present a case which occurred as a result of excessive manipulation by an unskilled birth attendant.

## 2. Case Report

**Figure 1:**
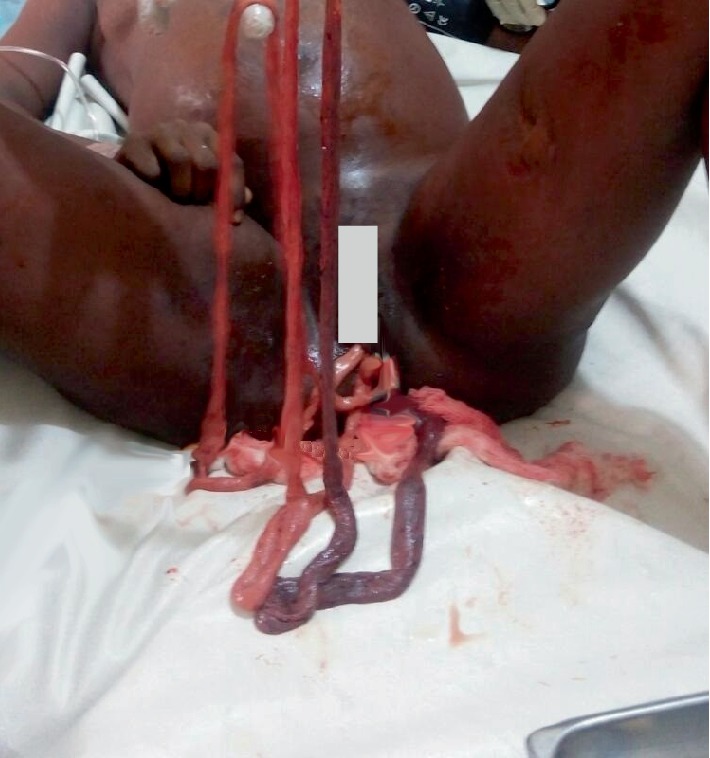
Extruded bowel loops through the vagina.

This was a case of a 23-year old G4 P3 A3 woman at a gestational age of 39 weeks in April, 2017 who was referred to the labour ward of the University of Calabar Teaching Hospital (UCTH), Calabar, Southern Nigeria, with a history of inability to deliver her baby after 5 days in labour prior to presentation. The patient had been managed in two different traditional birth attendant (TBA) homes. The second TBA recognized that the cause of prolonged labour was abnormal lie of the fetus and attempted to correct the lie by intrauterine manipulation through the vagina. This resulted in uterine rupture and subsequent evisceration of the intra-abdominal viscus through the vagina. The loop of the small intestine was initially mistaken for umbilical cord by the TBA who pulled several lengths of it through the vagina (Figure 1). Following the failed attempt to deliver the baby and the woman's deteriorating clinical state, the patient was subsequently rushed to UCTH.

At UCTH, the patient was resuscitated. Parenteral broad spectrum antibiotics were administered. The extruded loops of bowel were wrapped in sterile guaze soaked with warm normal saline. A general surgeon was invited to take part in the management of the patient. The patient was immediately prepared and taken to theatre for emergency laparotomy.

A midline incision was made to access the peritoneal cavity. Intra-operatively, a macerated female fetus was found in the uterine cavity. There was a left postero-lateral uterine wall tear extending from the mid portion to the posterior vaginal fornix. The loops of bowel extruded through this opening. The dead fetus was extracted. About 1,200 ml of blood in the peritoneal cavity was suctioned. Total abdominal hysterectomy was performed.

**Figure 2:**
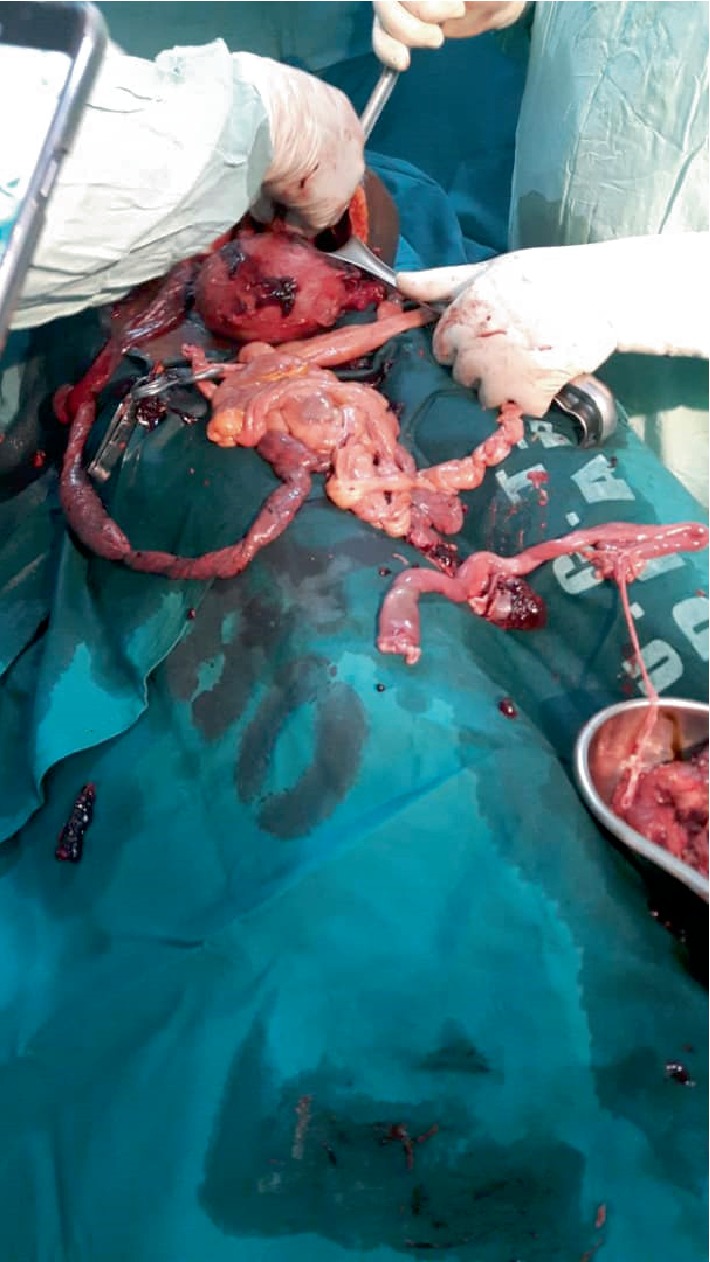
Ruptured uterus with devitalized intestine.

**Figure 3:**
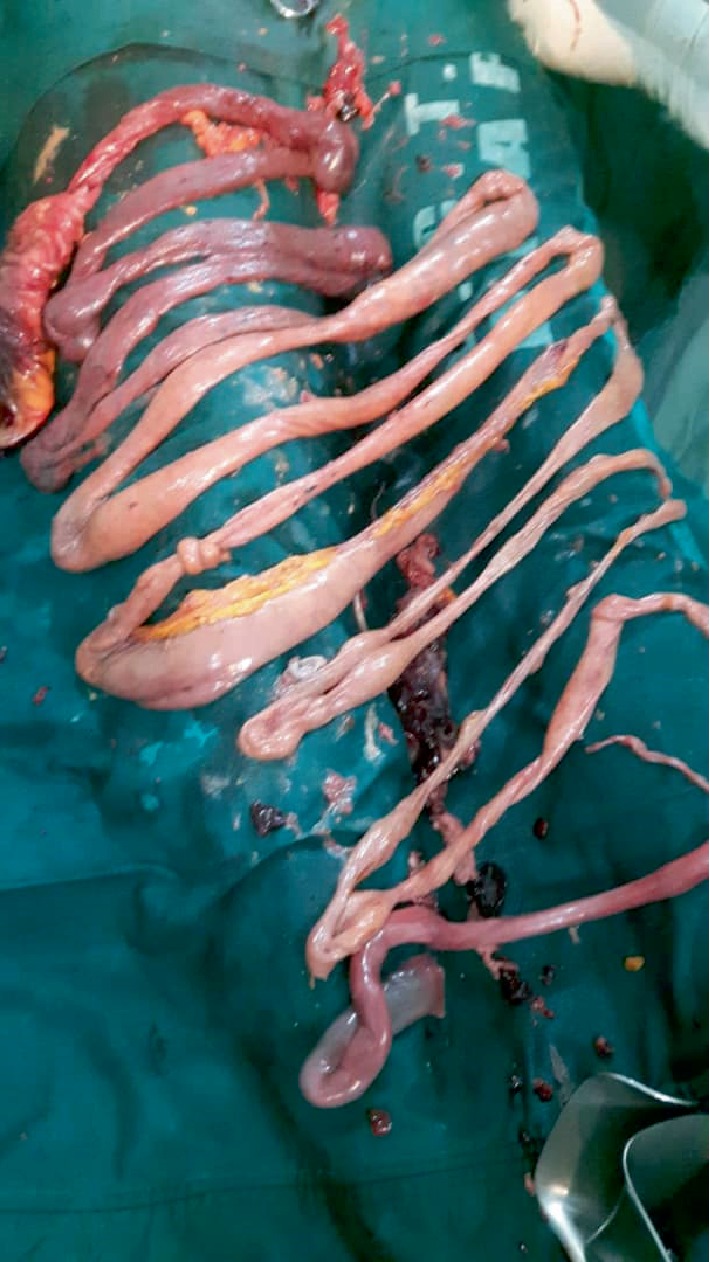
Resected devitalized intestine.

The whole intestines were thoroughly examined (Figures 2 and 3); about half the length of the intestine was devitalized and therefore, resected and an end-to-end anastomosis was done. The peritoneal cavity was lavaged with warm normal saline before abdominal wall closure. Nasogastric tube was then inserted.

Post-operatively, the patient was given parenteral nutrition for 5 days. Antibiotics, analgesics and other supportive treatments were continued according to our protocols. The patient opened bowel on the eighth post-operative day. The patient was commenced on graded oral meals which the patient tolerated. Subsequent post-operative days were uneventful. The patient was later discharged home.

## 3. Discussion

This case highlights the grave danger of unsafe delivery especially in the hands of unskilled birth attendants. It may be difficult to assess the positive impacts of the traditional birth attendants in maternal care. In 2010, the government of the republic of Zambia discontinued the training of traditional birth attendants and banned them from conducting deliveries as they were viewed as contributing to maternal mortality [7]. A follow-up study evaluating the positive impact of such bold government decision revealed that there was remarkable improvement in maternal health which included early detection of complications and increased hospital supervised childbirths, enhanced clean and safe delivery as well as HIV/AIDS prevention [8]. Evidently, this led to a significant reduction in maternal mortality and morbidity. There may be need for gradual replacement of TBAs with skilled birth attendants in local communities in Africa.

It may be a big challenge in some African countries as many uneducated women in low-resource settings still prefer delivering under the care of TBAs as this is our index patient. This constitutes unsafe delivery and a serious challenge to safe motherhood initiative. As their activities are difficult to stop completely in our communities, training of TBAs should be gradually phased out and replaced with skilled birth attendants.

In conclusion, this case highlights that when uterine rupture occurs, any cord-like structure protruding per vaginum may not always be an umbilical cord. There is need for gradual replacement of TBAs by skilled birth attendants in our local community if we are to improve the outcome of maternal care and work towards the attainment of sustainable development goals.
